# Sensitivity Analysis of an Individual-Based Model for Simulation of Influenza Epidemics

**DOI:** 10.1371/journal.pone.0045414

**Published:** 2012-10-29

**Authors:** Elaine O. Nsoesie, Richard J. Beckman, Madhav V. Marathe

**Affiliations:** 1 Network Dynamics and Simulation Science Laboratory, Virginia Bioinformatics Institute, Virginia Tech, Blacksburg, Virginia, United States of America; 2 Department of Computer Science, Virginia Tech, Blacksburg, Virginia, United States of America; Northeastern University, United States of America

## Abstract

Individual-based epidemiology models are increasingly used in the study of influenza epidemics. Several studies on influenza dynamics and evaluation of intervention measures have used the same incubation and infectious period distribution parameters based on the natural history of influenza. A sensitivity analysis evaluating the influence of slight changes to these parameters (in addition to the transmissibility) would be useful for future studies and real-time modeling during an influenza pandemic.

In this study, we examined individual and joint effects of parameters and ranked parameters based on their influence on the dynamics of simulated epidemics. We also compared the sensitivity of the model across synthetic social networks for Montgomery County in Virginia and New York City (and surrounding metropolitan regions) with demographic and rural-urban differences. In addition, we studied the effects of changing the mean infectious period on age-specific epidemics. The research was performed from a public health standpoint using three relevant measures: time to peak, peak infected proportion and total attack rate. We also used statistical methods in the design and analysis of the experiments.

The results showed that: (i) minute changes in the transmissibility and mean infectious period significantly influenced the attack rate; (ii) the mean of the incubation period distribution appeared to be sufficient for determining its effects on the dynamics of epidemics; (iii) the infectious period distribution had the strongest influence on the structure of the epidemic curves; (iv) the sensitivity of the individual-based model was consistent across social networks investigated in this study and (v) age-specific epidemics were sensitive to changes in the mean infectious period irrespective of the susceptibility of the other age groups. These findings suggest that small changes in some of the disease model parameters can significantly influence the uncertainty observed in real-time forecasting and predicting of the characteristics of an epidemic.

## Introduction

Sensitivity analysis is the study of the contribution of different parameters to the uncertainty present in the outcome of a system [1], [2]. Various scientific fields use sensitivity and uncertainty analysis to: (i) highlight important and remove irrelevant data, (ii) optimize the design of a system and (iii) rank by importance the influence of various parameters on the behavior of a system [3], [4]. The scope of a sensitivity analysis procedure can be local or global. Local analysis aims to examine the effects of local deviations of a parameter or a chosen trajectory in the parameter space [5]. Alternatively, global analysis is used to evaluate the entire parameter space in addition to interactions between parameters to determine all of the system's critical points [3], [6]. Methods for sensitivity analysis can be either statistical or deterministic [2], [7]. However, selection of methods depends on the purpose and system under study. Typically, complex systems (models) are computationally expensive which tends to limit the scope of a sensitivity analysis.

In this study, we perform sensitivity analysis on a complex individual-based stochastic epidemiology model for the study of influenza epidemics. Individual-based models are increasingly used in the study of the dynamics of infectious diseases and evaluation of methods for controlling the spread [8]. For a few examples, see [9], [10] and [11]. These models capture human-to-human disease transmission by creating synthetic populations with time-varying contact networks [12]. The level of detail used in these models increases the complexity but also enables the model to more realistically capture the heterogeneity present in the natural system [8]. Although the level of realism is beneficial, the behavior of the systems can be challenging to explore analytically due to the large number of parameters [2]. In addition, validation of parameters used in these models tends to depend on qualitative comparison of model behavior and expert opinion [13].

In several studies, the sensitivity analysis of individual-based models of epidemic dynamics have been used to evaluate the effect of disease parameters on public policy related questions. For example, for a model aimed at simulating Smallpox epidemics, the sensitivity analysis could focus on model assumptions relating to how changes in individual behavior after infection might influence the observed outcomes such as in the study by Burke et al. [14]. For individual-based models used in the study of influenza-like epidemics, the sensitivity analysis could focus on changes in interventions and response strategies such as in the studies by Halloran et al. [15] and Germann et al. [16]. In this study we aim to explore the sensitivity of an individual-based epidemiology model to changes in the assumptions made regarding the characteristics of the disease. The disease model is one of the two major components of the individual-based model. The other is a state-of-the-art behavioral model which consists of synthetic populations and time-varying social networks. There have been several studies validating the structural aspects of the individual-based model [15], [17], [18] and the sufficiency of the amount of detail used in its development [19]. However, there have not been any studies exploring the epidemiological and mathematical assumptions relating to the underlying process describing disease transmission.

### Parameters

**Figure 1:**
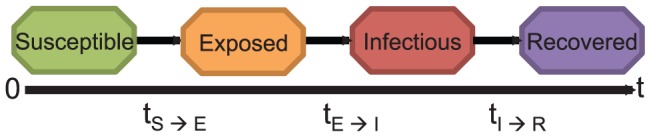
The SEIR disease model used in describing disease progression within the individual-based model. Individuals move through four health states. Susceptible individuals become exposed to the disease due to contact with an infected individual. After being exposed, an individual becomes infectious. An infectious person recovers at the end of the infectious period. Recovered individuals can no longer spread the disease.

We perform sensitivity analysis on the parameters of a networked **S**usceptible-**E**xposed-**I**nfectious-**R**ecovered (SEIR) disease model. The four disease states in the SEIR model (Figure 1) are used in describing within host disease progression and between host influenza transmission in the social network [12]. To simplify the disease process, three parameters are used: transmissibility, incubation period distribution and infectious period distribution. The transmissibility is the diffusion intensity of a disease through a population. The transmissibility is usually measured using the reproductive number - the number of secondary cases for each primary case. The incubation period is the interval during which infected individuals cannot spread the disease and usually lasts between 1–4 days for seasonal influenza [20]. The infectious period duration is the period during which infected individuals can transmit the disease to susceptible individuals. During typical seasonal influenza epidemics, infectious individuals can shed the virus a day before onset “through 5–10 days after illness onset” [20]. In this model, the incubation and infectious periods are described using discrete probability distributions since individuals in the population tend to have different incubation and infectious period durations based on their age and health status. The initial (base case) parameters based on the natural history of seasonal influenza have been used in several studies [15], [19], [21], [22]. The base case incubation period distribution is defined as follows: 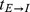 = 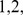 or 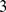 days with probability 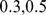 or 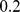, respectively. This implies an infected individual can have an incubation period duration of 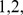 or 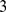 days with probability 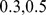 or 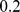. Likewise, the infectious period distribution is given by: 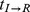 = 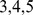 or 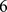 days with probability 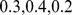 or 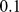, respectively. To our knowledge, there is no defined standard for performing sensitivity analysis on parameters which are non-parameterized discrete distributions (in contrast to parameterized distributions like the Poisson or the binomial), especially not in individual-based models. Therefore, we use a combination of statistical methods and present a sensitivity analysis study which provides a framework for future studies.

### Aims and Relevance

This study is motivated by the need to improve methods for real-time modeling and predicting during a pandemic [23]. The usefulness of real-time modeling was illustrated during the 2009 influenza pandemic using both compartmental models [24] and individual-based models [25]. To improve real-time epidemic modeling using individual-based models, we need an in-depth knowledge of the effects of the disease parameters on the dynamics of predicted epidemics. We therefore explore the following aims: (i) evaluate individual and joint effects, and rank parameters based on influence on simulated epidemics and (ii) compare the sensitivity of the model across age groups and social networks with demographic differences. Studies have indicated that differences in the transmission of the 2009 H1N1(A) virus in various regions was partly due to differences in population demographics [26], . In addition, several studies have suggested that school children tend to influence the propagation of influenza [28]–[31]. Both observations are further investigated in this study under different parameter combinations. Comparison of the sensitivity across networks with demographic and urban-rural differences is essential since this would indicate whether results observed for one social network are reproducible in another. The experiments and analysis are expected to further our knowledge of how to improve real-time modeling of epidemics using such models.

A good understanding of how the disease parameters influence the dynamics of simulated epidemics would aid in the prediction of the epidemic curve and estimation of parameters during an epidemic of a novel influenza virus. The epidemic curve for the purpose of this study is the daily number of infected persons for the duration of an epidemic. There are several possible approaches for real-time estimation of disease parameters during an epidemic [24], [32], [33]. However, uncertainty in the data collected during an epidemic can result in extremely unreliable results [23]. In addition to improving input data used in prediction, a study of how minute changes in the model parameters affect the predicted outcomes would be invaluable. For instance, [21] proposed a real-time epidemic curve prediction method based on matching surveillance data for an ongoing epidemic to epidemics simulated using parameters from previous outbreaks. If the new epidemic cannot be matched to any of the simulated cases, then a combination of expert opinion and search algorithms are used in suggesting new parameters. A sensitivity analysis study would enable easy assessment of the initial values and selection of the parameter space for the search algorithm.

To accomplish these aims, we explore the space of possible incubation and infectious period distributions by generating distributions with the same mean and also by perturbing the probabilities of the base case distribution. This process is further discussed in the problem definition. We use mono-factorial (one-factor-at-a-time) and full factorial designs to study the effects of each parameter and joint effects due to interactions between parameters. We also use principal components clustering, analysis of variance, and Pearson correlation to determine the level of sensitivity in the model [2]. Moreover, we use the epidemic curve as the main outcome measure. These procedures and reasons for selecting them are discussed in later sections.

**Figure 2:**
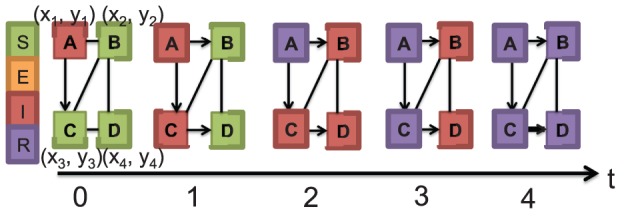
Example of SEIR model describing between host disease transmission. There are four individuals/nodes in the contact network and five edges. Different colors indicate different health states. Also each node is randomly assigned an incubation and infectious period 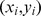 sampled from a discrete distribution. For simplicity, each of the nodes in this example has an incubation period of 0 days and an infectious period of 2 days. On day 0, node *A* is infectious while all other nodes are susceptible. On day one, node *C* is infected due to contact with node *A* and on day two, node *A* recovers, while nodes *B*, *C* and *D* are infectious. There are no susceptible nodes after day two. On day three, node C recovers. Finally on day four, all nodes recover. Unlike this example, the networks used in this study have approximately 76000 and 20 million nodes.

The complexities of this study lie in quantifying the sensitivity of an individual-based model to changes in parameters which are discrete probability distributions. As shown in Figure 2, each individual in the population is randomly assigned an incubation and infectious period duration sampled from a probability distribution. For each replicate of an epidemic simulation, individuals receive new samples from the incubation and infectious period distribution. Variations in these parameters add to the stochasticity present in the model. Unlike most studies where the difficulty of sensitivity analysis is introduced by the number of parameters, in this study the complexity lies in the model and type of parameters. To our knowledge, no previous studies have focused on quantifying the sensitivity of an individual-based model to changes in parameters which are discrete probability distributions.

### Overview of the Individual-based Model

The process of developing the individual-based model is described briefly since it is not a novel contribution of this work. A more detailed description of the individual-based model can be found in [12] and the supporting information. The individual-based epidemiology model consists of the social network and the disease model. The disease model is described in the next section. The model includes representation of each individual in a population along with activity schedules describing their movements. Contacts that occur between individuals as they move about their daily schedule are represented using time-varying contact networks. These contacts can result in disease transmission depending on the duration, type of contact and health state of the individuals. The main steps involved in the construction of the individual-based model are: creation of the synthetic population; activity and location assignment; the definition of an infectious disease model and interventions both pharmaceutical and non-pharmaceutical used in controlling the propagation of the disease.

The synthetic population is created using demographic information, survey and land use data. Synthetic individuals are defined with specific sets of demographic variables and assigned to households based on US census data provided in SF3 and PUMA (Public Use Microdata Area) files [34]. Each individual is assigned to a household. Some of the demographic information available for each individual in the synthetic population includes age, education level, and household income. The synthetic populations are created to represent the true population as realistically as possible, while maintaining confidentiality. A census collected at the block level of the synthetic population is statistically indistinguishable from the census data [17]. Individuals in the synthetic population interact with each other and their environment to produce time-varying social contact networks. Further information on the creation of the synthetic social network can be found in [17], [35] and [36].

In addition to having specific demographic information, each node in the synthetic population is also allocated activities based on thousands of responses to a time-use survey for a specific geographical region. As expected, there will be differences in the time-use survey collected in different geographical regions such as New York City (and surrounding metropolitan regions) and Montgomery County in Virginia due to demographic differences (hereafter referred to as New York and Montgomery County). The National Household Transportation survey is used in this model to create the activity templates. Activities can include shopping, work, daycare, etc. Individuals in the synthetic populations are matched to the survey households using decision trees based on demographic variables. Activities are then assigned realistic geographical locations using a gravity model based on land-use data [17]. In addition, each activity is assigned a start and end time, resulting in a minute-by-minute schedule for each synthetic individual. Currently, this modeling approach is considered the *de facto* standard for travel demand models in transportation science [11].

The social contact network results from interactions between individuals in the synthetic population based on their activity schedules. Individuals are represented as vertices in a graph and edges are used to describe interactions between individuals (Figure 2). The modeling approaches used in each step of creating the individual-based model can be found in [11] and the supporting information. Additional information can also be found in [37] and [38].

### Disease Model

Using a computational model such as the basic SEIR model (Figure 1), disease transmission is explained within the previously described network. This implies that each individual moves through four disease states (susceptible, exposed, infectious and recovered), and transmission is dependent on the contact between two individuals in the susceptible and infectious states. The transition between disease states is probabilistic and timed (e.g. represented by a probability distribution), and can also depend on the demographics of an individual (such as their age, work and health status). As individuals go about their different activities (such as shopping, and work) they come in contact with other individuals. Through this process, the disease can be transmitted from an infected individual to a susceptible individual. See (Figure 2) for an example illustrating disease transmission between nodes in a basic network.

For the disease model used in this study, the probability that an infectious person *i* infects a susceptible person *j* is given by:
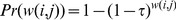
(1)where 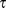 is the probability of transmission per unit of contact time between persons *i* and *j* [12]. *w(i,j)* is the contact duration between *i* and *j* measured in seconds. The SEIR model is one of the simplest disease models which can be used for this individual-based model. Additional information can be added to the disease model to better capture different infectious diseases. In addition, intervention options such as vaccination, antiviral and social distancing are included in the model to control disease spread. Single interventions or a combination of interventions can be introduced either at the start or during a simulated epidemic.

Simulations are run by randomly selecting a number of people to introduce the disease into the population. During each simulation, we keep track of information on all contacts, duration of contact, and which contacts result in infection. Information on the contacts resulting in disease transmission, the vulnerability of the nodes, the epidemic size and epidemic curve are some of the information used in studying the dynamics of an epidemic.

### Problem Definition

The following formulation is used in defining the problem. Given a distribution 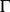: a vector with finite support 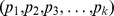 where 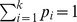: 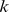 is the number of days and 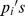 are the probabilities of observing each day. In terms of the incubation (infectious) period distribution, 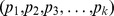 are the probabilities that an infected (infectious) individual will have an incubation (infectious) period of 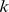 days.

There are several possible techniques for generating new distributions depending on the aim of the study. A likely procedure would involve placing a probability distribution such as the beta density [39] over 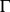. New distributions can be created by changing the shape parameters. This implies that a new distribution for the incubation and infectious period is defined by systematically generating new vectors, 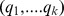 such that 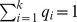. If this process is carried out naively, an infinite number of possible distributions can be generated. A simple approximation is to use a step size *d* to perturb one 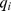 to another. The mean of the distribution is shifted by a few days using such perturbations. This method can be used to study how changes in the mean of the incubation and infectious periods affect the behavior of the model.

An alternative to the previously described procedure would involve defining the mean duration of incubation or infectiousness while randomly generating new distributions with different variances. This technique would enable the study of the effects of the variance of the incubation and infectious period distributions on the dynamics of the epidemics. In addition, the random generation of distributions would result in distributions with different shapes which might not be epidemiologically relevant for influenza. However, this allows for a mathematically exploration of the parameter space. It also enables the applicability of this procedure to models of other infectious diseases with parameters that are distinct from those based on the natural history of influenza. In this study, we use both perturbations of the 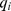 *s* and random generation around the same mean. We selected these procedures based on their simplicity and results from preliminary studies which indicated that these techniques are sufficient for investigating the aims in this study.

The above procedures are for the incubation and infectious period distributions only. For the transmissibility, which is a real number, values are selected to simulate epidemics similar to seasonal influenza, previous pandemics and more extreme epidemics.

To perform sensitivity analysis on these three quantities, we explore the mapping 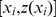, 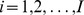, where 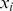 represents the parameters: transmissibility, infectious period distribution, and the incubation period distribution [2]. 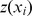 are the epidemic curves resulting from different parameter combinations. See Table 1 for a summary of the notations used in this study.

**Table 1:** Table of notations.

Notation	Description
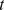	time
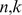	counts
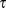	unit of contact time
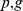	probability
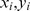	factor
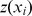	epidemic curves

Sensitivity analysis involving non-parameterized discrete distributions present special problems that inhibit the uses of standard techniques such as Latin Hypercube Sampling [40]. The difficulty arises due to the mathematical dependence between probabilities representing the distribution (e.g. the sum of any two probabilities must be less than or equal to 1). Therefore we did not consider using such a design.

## Methods and Analysis

We used statistical methods and tests in the design and analysis of the experiments in this study. Since no statistical methods are universally accepted as infallible, we chose methods based on their applicability to the study [6]. As previously mentioned, for the study design we used factorial experiments [41]. Mono-factorial experiments were used in studying the sensitivity of the model to each of the parameters. A full factorial design was used in evaluating the influence of factor interactions on the observed outcomes. In addition, to find underlying groupings within the collection of curves from all factorial experiments, we used principal components clustering [42]. The groupings of curves with similar structures aided in determining the influence of the different parameters on the shape and form of the epidemic curves. These procedures are described below.

### Public Health Measures

**Figure 3:**
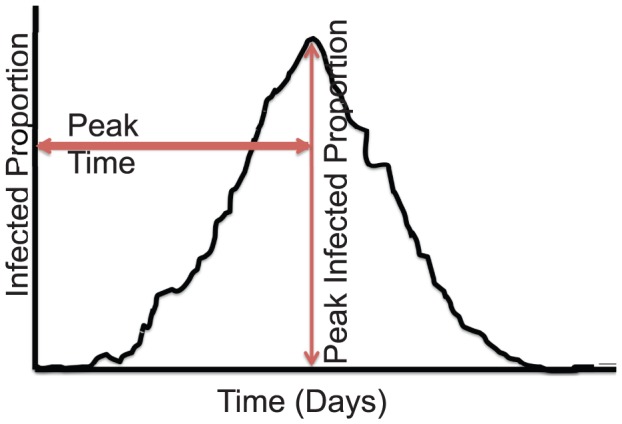
Public health measures (peak infected proportion, peak time and total attack rate) used in comparing epidemic curves.

As earlier stated, the epidemic curves were used as model outcomes 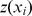. To facilitate comparison of the epidemic curves, we performed the analysis from a public health standpoint using three relevant measures: peak infected proportion, time to peak or peak time and total attack rate or total infected proportion as shown in Figure 3. The *attack rate = infected-counts/population size*. The peak infected proportion indicates the point of maximum demand of public health resources such as nurses and hospitals during an epidemic. The peak time suggests the available time for implementing control measures such as vaccines, antivirals, and sequestration to achieve optimal effectiveness. Lastly, the total attack rate can be used to quantify the disease's effect based on the morbidity and mortality rates [19], [43]. These measures are also useful for real-time forecasting and prediction of the impact of an epidemic.

Analysis of variance (ANOVA) was used to evaluate differences in the mean of the public health measures given changes in the parameters. In its simplest form, ANOVA is an extension of the t-test. ANOVA is typically used to test whether the means of several groups are equal. The groups in this study were the different sets of epidemics/experiments resulting from changes in the parameters. We tested whether the means of the various peak infected proportions, peak times and attack rates for the different sets of epidemics were equal. If a statistically significant difference was observed, we used a pairwise t-test with Bonferroni correction to find which pairs of experiments were statistically different.

### Social Networks

We used two synthetic social networks in order to compare possible effects of demographic and rural-urban differences. The demographic information for the synthetic populations in the social networks were based on the population of Montgomery County and New York with approximately 76000 and 20 million synthetic individuals respectively. The network for Montgomery County is available at http://ndssl.vbi.vt.edu/opendata/index.php. The same disease model parameters were used in simulating epidemics over the synthetic social networks. As previously mentioned, random sampling was used in the assignment of the incubation and infectious period to the nodes in the network. Random assignment was selected instead of targeted assignment because it is simpler to implement, and increases the flexibility of designing and running experiments. In addition, random assignment of these parameters has been used in most studies [10], [11], [19], [21], [22].

### Factorial Experiments

In the mono-factorial design, we varied a single parameter and held the others fixed. In the first set of experiments, we altered the incubation and infectious period parameters by shifting the mean by a single day. The main reason for doing this was to evaluate how changes in the mean infectious and incubation period distribution would affect the dynamics of simulated epidemics. This was investigated for two reasons: (i) the natural history of infection for the 2009 H1N1(A) pandemic was similar to that for seasonal influenza [44] and (ii) typically the mean incubation and infectious period are reported during an influenza epidemic, not the entire distribution.

We varied each of the parameters over five values and studied the effects of these changes to the dynamics of the simulated epidemics. First, using five transmissibility values, one incubation period distribution and one infectious period distribution, we simulated five sets of epidemics. The five transmissibility values were in the range: 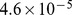 to 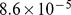 per sec/contact time. As earlier mentioned, these values were selected to simulate epidemics similar to previous pandemics and more extreme outbreaks. Next, to evaluate the effects of the infectious period distribution on the epidemic curve, we experimented with five infectious period distributions, a fixed transmissibility and one incubation period distribution. The mean of the infectious period was altered across two to six days. Recall that the mean of the base case infectious period distribution was four days. So the new distributions either had a mean that was greater or less by a day or two compared to the base case. Finally, to evaluate the effects of the incubation period, we generated five incubation period distributions while holding the transmissibility and infectious period distribution fixed. The mean incubation period fluctuated between one to five days. All epidemics were simulated over both social networks. In addition, each epidemic was replicated twenty-five times. These experiments are labeled as Exp.1–3 in Table 2. The results from this set of analyses led to the second set of experiments.

**Table 2:** Summary of Experimental Design.

Labels	Experiment Description	Transmissibility	Incubation Period	Infectious Period
			Distribution	Distribution
Exp.1	Mono-factorial experiment	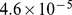	1:0.3 2:0.5 3:0.2	3:0.3 4:0.4 5:0.2 6:0.1
	with focus on slight changes	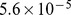		
	to the transmissibility	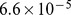		
		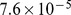		
		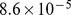		
Exp.2	Mono-factorial experiment to	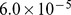	1:0.3 2:0.5 3:0.2	3:0.3 4:0.4 5:0.2 6:0.1
	evaluate the effect of changes		0:0.3 1:0.5 2:0.2	
	to the mean of the incubation		2:0.3 3:0.5 4:0.2	
	period distribution		3:0.3 4:0.5 5:0.2	
			4:0.3 5:0.5 6:0.2	
Exp.3	Mono-factorial experiment to	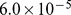	1:0.3 2:0.5 3:0.2	3:0.3 4:0.4 5:0.2 6:0.1
	investigate the effect of changes			2:0.3 3:0.4 4:0.2 5:0.1
	to the mean of the infectious			1:0.3 2:0.4 3:0.2 4:0.1
	period distribution			4:0.3 5:0.4 6:0.2 7:0.1
				5:0.3 6:0.4 7:0.2 8:0.1
Exp.4	Mono-factorial experiment to	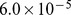	1:0.3 2:0.5 3:0.2	3:0.3 4:0.4 5:0.2 6:0.1
	study the effect of fixing the		1:0.2 2:0.5 3:0.3	
	mean while changing the		1:0.3 2:0.4 3:0.3	
	variance and shape of the		1:0.325 2:0.45 3:0.225	
	incubation period distribution		1:0.25 2:0.40 3:0.35	
			1:0.3335 2:0.333 3:0.3335	
			1:0.35 2:0.3 3:0.35	
			1:0.4 2:0.2 3:0.4	
			1:0.375 2:0.25 3:0.375	
			1:0.333 2:0.334 3:0.333	
			1:0.2 2:0.4 3:0.4	
			1:0.233 2:0.3 3:0.467	
Exp.5	Mono-factorial experiment to	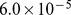	0:0.223 1:0.405 2:0.372	2:0.1 3:0.2 4:0.3 5:0.4
	study the effect of fixing the		0:0.123 1:0.605 2:0.272	2:0.15 3:0.2 4:0.3 5:0.2 6:0.15
	mean while changing the			2:0.1 3:0.2 4:0.4 5:0.2 6:0.1
	variance and shape of the			3:0.2 4:0.6 5:0.2
	infectious period distribution			3:0.13 4:0.74 5:0.13
				2:0.2 3:0.2 4:0.2 5:0.2 6:0.2
				3:0 4:1 5:0
				3:0.17 4:0.66 5:0.17
				3:0.1 4:0.8 5:0.1
				3:0.4 4:0.2 5:0.4
				3:0.5 4:0 5:0.5
				3:0.25 4:0.5 5:0.25
				3:0.333 4:0.334 5:0.333
Exp.6	Full factorial experiment to	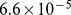 [t1]	1:0.3 2:0.5 3:0.2[inc1]	3:0.3 4:0.4 5:0.2 6:0.1 [inf1]
	investigate the impact of	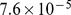 [t2]	3:0.3 4:0.5 5:0.2[inc2]	2:0.3 3:0.4 4:0.2 5:0.1 [inf2]
	interactions between parameters	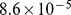 [t3]	2:0.3 3:0.5 4:0.2[inc3]	1:0.3 2:0.4 3:0.2 4:0.1 [inf3]
	and rank parameters by			
	influence			

The experimental description indicates the type of statistical design and the parameter under focus. We present the incubation and infectious period distributions as follows: 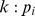 where 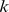 is the day and 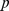 the probability.

In the second set of experiments, we further assessed the effects of the mean and variance of the incubation and infectious period distributions on the dynamics of an epidemic. To evaluate the effects of the variance of the infectious period duration on the dynamics of the simulated epidemics, we randomly created thirteen infectious period and twelve incubation period distributions. The infectious period distributions were defined with possible infectious durations between two and six days. We randomly assigned probabilities to observing each day such that the mean infectious period was four days. Similarly, we also defined incubation period distributions with possible incubation durations between one and three days. The probabilities of observing each of these days in the synthetic population was defined such that the mean incubation period was two for all distributions.

We simulated twenty-six epidemics using each of the infectious period distributions, two similar incubation period distributions and one transmissibility. Each epidemic was replicated twenty-five times. These experiments are labeled Exp.5 in Table 2. Next, to study the influence of the variance of the incubation period distributions on the dynamics of simulated epidemics, we simuated twelve epidemics with twenty-five replicates each (Exp.4 in Table 2). Each epidemic was simulated using one of the incubation period distributions, a single transmissibility and one infectious period distribution. The mean of the incubation and infectious period distributions were set at two and four days respectively because those were the means of the base case distributions [15]. We analyzed how the variance of these distributions affected the dynamics of the epidemics. The simulated epidemics were compared based on the results from an ANOVA and t-test with Bonferroni correction for multiple comparisons. We tested for significant differences in the mean of the total attack rate, peak infected proportion and time to peak.

Using a full factorial design, we varied each of the parameters across three levels, resulting in twenty-seven combinations/experiments. The parameters used in the full factorial experiments were generated by shifting the mean of the base case distributions by a single day. This resulted in infectious period distributions with means of three, four and five days. The incubation period distributions had a mean of one, two and three days. This variation in the parameters was done for the same reason as the first set of experiments. Each parameter was defined at three levels as shown in Exp.6 in Table 2. The parameter levels were labeled: (t1, t2, t3), (inc1, inc2, inc3) and (inf1, inf2, inf3). The epidemics each had fifty replicates and were simulated for a duration of three hundred and fifty days so as to accommodate epidemics with durations longer than the typical influenza season in the United States. The epidemics were analyzed using ANOVA and clustering with principal components.

As previously stated, due to observations made during the 2009 H1N1 pandemic, we also evaluated the sensitivity of the model based on disease spread within age groups. The four age groups were: pre-schoolers, school-agers, adults and seniors. The epidemics were simulated using the parameters for Exp.3 in Table 2 because the full factorial analysis indicated that the infectious period distribution had the strongest influence on the epidemics compared to the other parameters. There was one transmissibility, one incubation period distribution and five infectious period distributions. The infectious period distributions had different mean durations ranging from two to six days. We evaluated how differences in the mean infectious period duration affected the dynamics of the age-specific epidemics. For each of the age-groups, we compared the time to peak, total and peak infected proportions. We performed this analysis under three scenarios. In the first case, we assigned the same susceptibility to all age-groups. In the second scenario, children and elderly were allowed a higher susceptibility. In the third case, only children had a higher susceptibility compared to all other age groups. These settings were modeled based on observations made during seasonal influenza epidemics and the 2009 H1N1 pandemic. In addition, several studies have suggested that school-age children are highly susceptible to infectious disease spread due to regular incidence of close proximity interactions [30]. We therefore studied how the epidemic dynamics for each age group (especially children) varied with changes in the infectious period distribution and age-specific susceptibility.

All experiments were implemented in the individual-based model under the base case scenario: no interventions were introduced to control the spread of the epidemic. To capture the heterogeneity present in the model and elucidate the influence of the social networks, each epidemic was replicated between 25 to 200 times. See Table 2 for a summary of the parameters used in these epidemics and Table 3 for a summary of the various components involved in the analysis.

**Table 3:** Summary of the Various Components in the Analysis.

Components	Description
Social Networks	Montgomery County (VA) and New York City
Factorial Experiments	Mono-factorial experiments and
	one full factorial experiment
Public Health Measures	Peak infected proportion(peak infected proportion),
	time to peak (peak time)
	and total attack rate (total infected proportion)
Statistical Methods	Analysis of variance (ANOVA), Pearson Correlation,
	T-tests with Bonferroni adjustment
	and Principal Components Clustering

### Principal Components Clustering

To uncover the parameter with the most significant influence on the structure of the epidemic curves, we used principal components clustering to find underlying groupings within the 1350 epidemic curves from the full factorial experiments. Typically, replicates of the same epidemic tend to have similar characteristics. Therefore, one would expect epidemics with the same parameters to fall into the same groups if clustered. However, this is not always the case due to the stochastic nature of the model and imperfection of clustering algorithms. In this study, we expected that by clustering the epidemic curves, we would observe patterns in the distribution of epidemics into different clusters based on the levels of the parameters. The parameter having the strongest influence on the structure of the epidemic curves should result in groupings based on different levels.

Epidemic curves can be viewed as time series since infected-counts are collected over fixed time intervals (e.g. on a daily or weekly basis). The daily infected-counts can be represented as a vector. The process of clustering based on principal component analysis was carried out as follows: first we estimated principal components based on the variance-covariance matrix of the vectors representing the epidemic curves. Next, we fit a linear regression equation to centered daily infected-counts for each epidemic curve to the nonlinear principal components. Lastly, the regression coefficients were clustered into groups using k-medoids, which is a robust version of the k-means algorithm. For additional information on this process see [42]. Several other clustering approaches could be used, however, the principal components clustering method was selected because the process captures curves with similar structures and the regression coefficients provide a description of the characteristics of curves within each cluster. The clustering was based on the first six principal components since those explained over 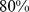 of the variance. Using additional components did not improve the clustering. We decided to use nine clusters after experimenting with different groupings. Increasing the number of clusters failed to provide better separation.

## Results

We present the results by outcome. Recall that the aims of the study are to: (i) evaluate individual and joint effects, and rank parameters based on influence on simulated epidemics and (ii) compare the sensitivity of the model across age groups and social networks with demographic differences. The results are presented only for New York since the observations are similar to that for Montgomery County as discussed in a later section.

### Finding 1. The transmissibility and mean infectious period duration significantly affects the peak time, peak infected proportion and total attack rate. In contrast, an increase in the mean incubation duration does not significantly influence the total attack rate, but slightly influences the peak time and peak infected proportion

#### Support

**Figure 4:**
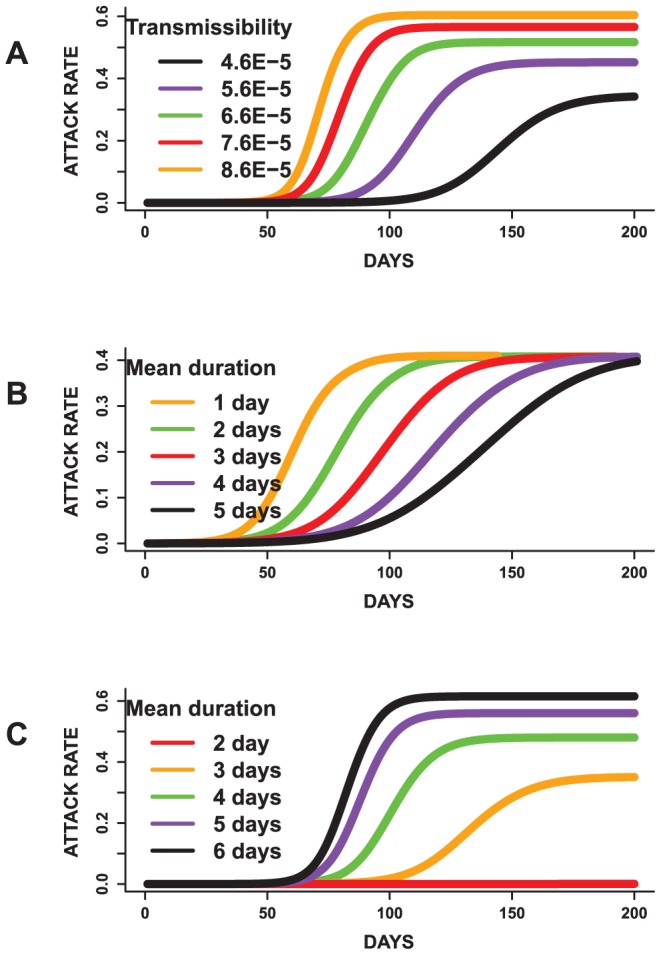
Results for mono-factorial experiments for each of the factors: (A) transmissibility, (B) incubation period distribution and (C) infectious period distribution. Each curve represents the mean attack rate over time based on 25 replicates of each simulation. (A) shows that an increase in the transmissibility increases the attack rate. (B) shows that changing the mean of the incubation period has a minimal effect on the total attack rate and (C) indicates that an increase in the mean infectious duration results in an increase in the attack rate. Simulations with mean infectious period of 2 days failed to become epidemics.

**Figure 5:**
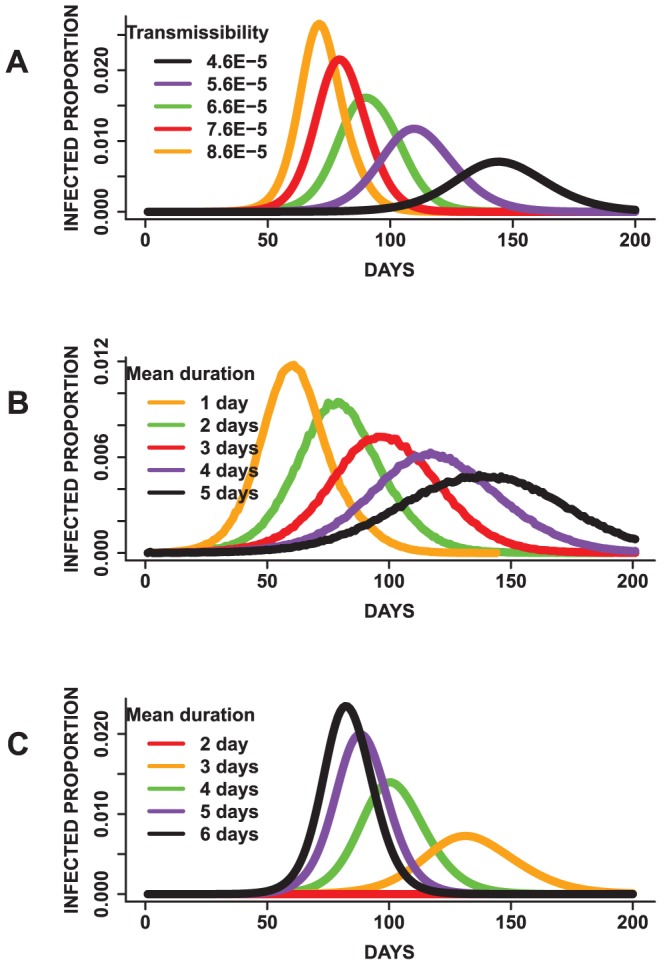
Results for mono-factorial experiments for each of the factors: (A) transmissibility, (B) incubation period distribution and (C) infectious period distribution. Each curve represents the mean epidemic curve from 25 replicates of each experiment. (A) Increasing the transmissibility shortens the peak time and increases the peak infected proportion. (B) A reduction in the mean infectious period results in earlier peaks and increases in the peak infected proportion. (C) An increase in the mean infectious duration results in shorter peak times and increases the peak infected proportion.

Figures 4 and 5 display mean epidemic curves from the first three mono-factorial experiments (Exp.1–3) in Table 2. Per Figure 4, increasing the transmissibility by a value of 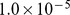 per sec/contact time raises the total attack rate by 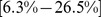. No overlap is observed between the ranges of the attack rates from the five sets of epidemics. A pairwise comparison using the t-test reveals statistical significant differences 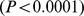 for all pairs.

Figure 4 also displays the results for the incubation and infectious period distributions based on the mean of the distributions. In general, raising the mean of the incubation period distribution does not significantly increase the variability observed in the attack rates. This is because an increase in the incubation duration does not affect the infectious duration. Although it takes a longer duration to become infectious, the time required to spread the disease is not affected, thereby resulting in similar attack rates. In contrast, an increase in the mean of the infectious period raises the total attack rate. Since more individuals have a longer infectious period, the disease tends to affect a larger proportion of susceptible individuals in the population.

The results for the peak time and peak infected proportion are displayed in Figure 5. Epidemics with a higher transmissibility tend to peak sooner, and have a higher morbidity rate compared to epidemics with a lower transmission rate. Pairwise t-tests to evaluate differences in the means of these measures suggests statistical significant differences 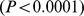. This indicates that the transmissibility has a major impact on the dynamics of the simulated epidemics, as would be expected. Though the attack rates for the incubation period experiments are not statistically significantly different per ANOVA, the mean peak time and peak infected proportions are statistically significantly different 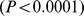. This implies that changing the mean incubation period does not significantly affect the total number of people infected but rather alters the shape of the epidemic curves.

Furthermore, changes in the infectious period distributions affects the peak time and peak infected proportion in a similar manner as the transmissibility. The peak infected proportion rises with an increase in the mean infectious period. Also, epidemics with higher mean infectious periods tend to peak earlier compared to epidemics with a lower mean infectious period. A pairwise comparison using the t-test indicates that the peak infected proportion, peak time and attack rates are statistically significantly different 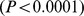 for all pairs.

Observations on the transmissibility and mean infectious period are not surprising. The basic reproduction number 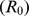 increases with an increase in the transmissibility with estimated mean values of 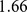, 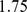, 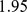, 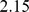 and 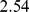 corresponding to transmissibility values of 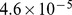 to 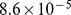 per sec/contact time. The mean 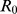 values are 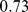, 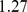, 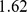, 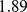 and 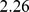 for mean infectious periods of 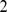, 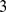, 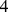, 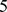 and 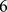 days respectively. However, no pattern is observed in the 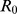 values for epidemics involving changes in the mean incubation period. The 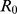 fell within a range of 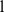 to 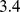 for replicates of each of the epidemics. The mean 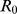 represents the mean initial effective reproduction number (effective 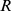). Plots of effective 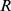 for Exp.1–3 are given in Figure S7.

These observations suggest that the mean infectious and incubation durations might have a role on the dynamics of the simulated epidemics. Therefore, we further investigate the effects of fixing the mean infectious and incubation periods as discussed in the next section.

### Finding 2. The mean of the incubation period distribution appears to be the sole determinant of its effects on the simulated epidemics. In contrast, the mean and variance of the infectious period distribution are needed to determine its influence on epidemic dynamics

#### Support

**Figure 6:**
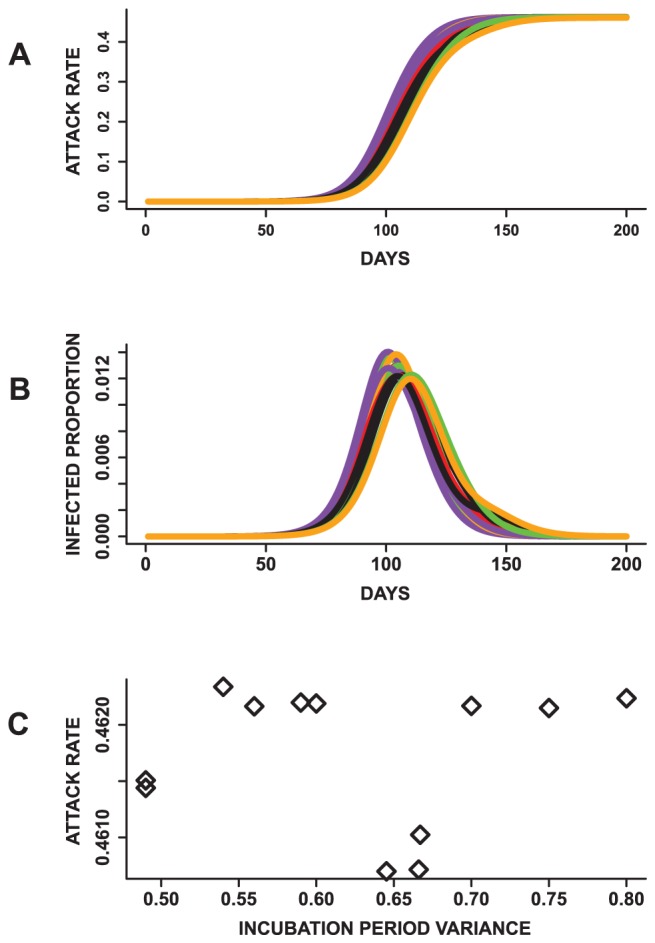
Results from mono-factorial experiments focused on changes in the variance of the incubation period distribution. (A) The mean total attack rates plotted over time. The total attack rates are not statistically significantly different across all 12 epidemics. (B) Epidemic curves showing the infected proportion over time. (C) A weak negative relationship is observed between the variance of the distributions and attack rates. These results indicate that epidemics with similar mean incubation periods can have similar attack rates even with differences in the variance of the distributions.

Figure 6 displays the mean epidemic curves resulting from simulated epidemics with similar mean incubation periods (Exp.4 in Table 2). The mean epidemic curves presented in Figure 6 are based on twenty-five replicates of each experiment. An ANOVA on the total attack rate indicates lack of statistically significant differences 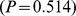. Similar results are observed for peak time and peak-infected proportions.

Moreover, a plot of the variance of the incubation period distribution against the mean attack rates (Figure 6) suggest a weak relationship with a Pearson correlation coefficient *r* of *−0.289*. A similar observation is also made between the variance of the incubation period distribution, mean peak time *(r = −0.263)* and mean peak infected proportions *(r = 0.146)* (Figure S1). This suggests that changes in the variance of the incubation period distribution does not have a strong influence on the total attack rate, peak time and peak infected proportions. These results also allude to the idea that solely changing the shape of the incubation period distribution while holding the mean incubation period fixed might not significantly influence epidemic prediction. Furthermore, changing the base case incubation period, which has been used in several studies [21], [22], [31], might not affect the results in these studies if the mean of the incubation period distribution remained the same.

**Figure 7:**
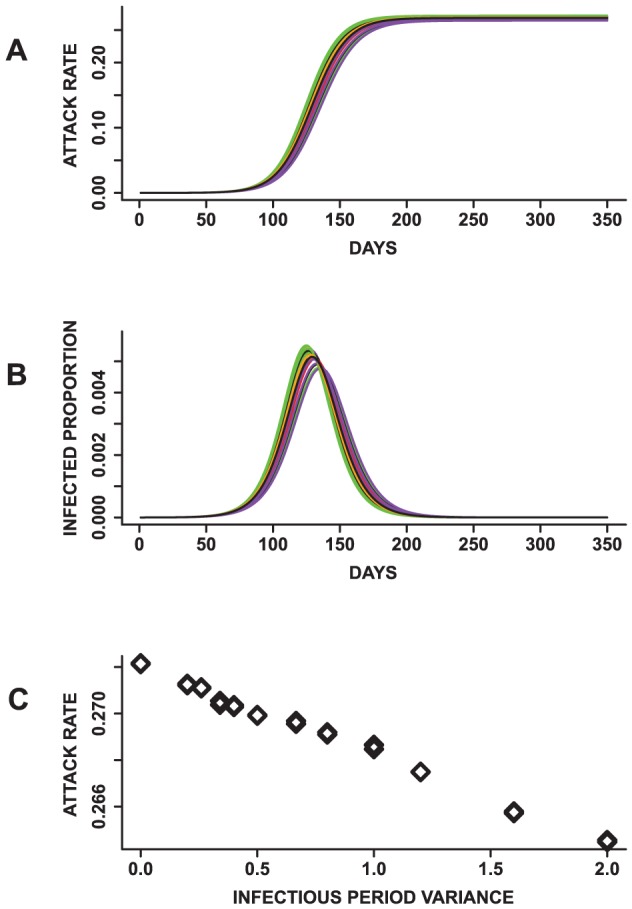
Results from mono-factorial experiments focused on changes in the variance of the infectious period distribution. (A) Mean attack rates for 24 epidemics with the same mean infectious period. (B) Mean epidemic curves showing daily infected proportion and (C) a plot of the infectious period variance against the attack rate. The means of the total attack rate, time to peak and peak infected proportion are all statistically significantly different based on an ANOVA and pairwise t-test. In addition, an increase in the variance results in a decrease in the total attack rate and peak infected proportion. These observations suggest that epidemics with the same mean infectious period can have different dynamics.

The results based on comparing epidemics resulting from infectious period distributions with the same mean values (Exp.5 in Table 2) are described in Figure 7 based on the mean epidemic curves. The epidemics appear to have similar shapes. An ANOVA indicates that at least one of the mean peak time, peak infected proportions and total attack rates is statistically significantly different 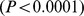 from the others. Since there are two incubation period distributions, we compare epidemics with the same incubation period distribution. The results are also significantly different for all public health measures.

In addition, a plot of the variance of the infectious period distributions against these measures suggests that the mean of the infectious period is not the sole influence on these measures. Rather, the total and peak infected proportions decrease with an increase in the variance of the infectious period distribution (*r = −0.99* and *−0.99*). On the contrary, the peak time increases with a raise in the variance of the infectious period distribution (*r = 0.95*) (Figure S2). These outcomes demonstrate that the dynamics of simulated epidemics might be sensitive to the mean, variance and shape of the infectious period distribution. These observations are contrary to that observed for the incubation period distribution where the mean of the distribution appears to be the sole determinant of its effects on the epidemic outcome.

### Finding 3. Compared to the other parameters, the infectious period distribution exerts the strongest influence on the total attack rate and structure of the epidemic curves

#### Support

The epidemics simulated based on the full factorial design (Exp.6 in Table 2) fail to have unique shapes (Figure S3). This indicates that a one-to-one mapping does not exist between the factor combinations and shape of the epidemic curves. In general, combinations of high transmissibility and long mean infectious periods result in epidemics which peak sooner and have a higher peak infected proportion. Epidemics with low transmissibility, long mean incubation periods and short infectious periods peak later and have lower peak infected proportions. Epidemics from all other factor combinations fall between. In addition, epidemics with the same transmissibility and infectious period distributions have similar mean total attack rates. For example, an ANOVA on the total attack rates of three sets of epidemics with the same transmissibility and infectious period distribution indicated a lack of statistical significant difference *(P = 0.871)*.

An ANOVA on the total attack rates, peak infected proportions, and peak times indicates that the mean of at least one of the experiments is statistically significantly different from the others 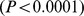. A pairwise comparison to find which experimental pairs are significantly different would have resulted in three hundred and fifty one comparisons. To simplify our analysis, we present the contributions to the variance of each of the measures in Table 4 and investigate the factor with the strongest impact on the structure of the epidemic curves.

**Table 4:** Analysis of contributions of the parameters to the variance observed in the total attack rate, peak proportion infected, and peak time.

Factor	Total Attack Rate	Peak Infected Proportion	Peak Day
Total Variance	25.36	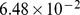	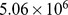
Transmissibility	2.659	0.0063	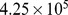
Infectious period	21.67	0.0511	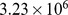
Incubation period	0.008	0.0058	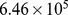

These are based on the full factorial design. The first row shows the total variance in each of the three outcome measures, while the rows beneath display the variance explained by each factor. Note that the infectious period explains the highest proportion of variance observed in all three public health measures.

**Figure 8:**
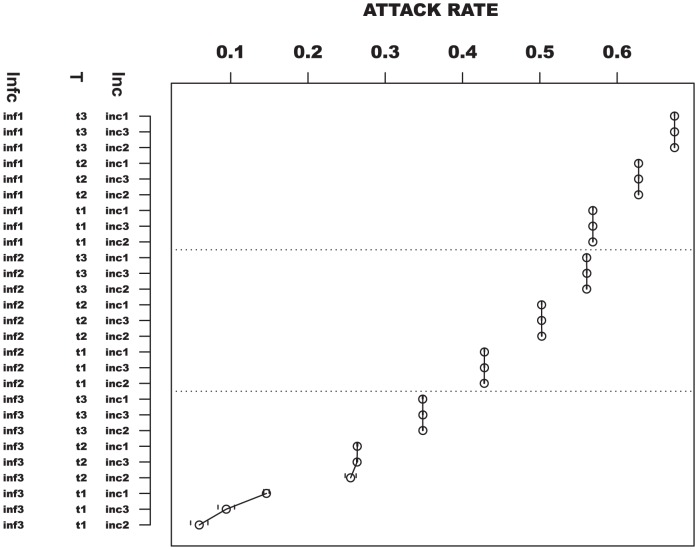
Plot of the mean total attack rate against all factor combinations. t stands for transmissibility, infc is an abbreviation for infectious period and inc represents the incubation period. The parameters are labeled according to Table 2. The factors are arranged by influence where the infectious period has the most influence on the total attack rate and the incubation period has the least influence on the total attack rate.

Per Table 4, the infectious period distribution explains the highest proportion of variance observed in the peak time, peak infected proportion and total attack rates. The parameters are ranked by variance explained in Figure 8. In terms of parameter combinations, transmissibility and infectious period distribution describes the highest proportion of variance for the total attack rate and peak time. On the other hand, the combination of the infectious and incubation periods explains the highest proportion of variance observed in the peak infected proportion (Table S1). However, the values noted for the interactions are much smaller than those for individual parameters. The results therefore indicate that the individual parameters explain most of the variance observed in the three public health measures.

To reaffirm the findings based on the ANOVA, we use principal components clustering to find underlying groupings within epidemic curves from all experiments. Epidemic curves from the twenty-seven experiments appear to be randomly distributed across clusters, irrespective of parameter combinations (Figure S4). Except for one cluster, there are more than two sets of parameter combinations in all the clusters. However, in most cases, the epidemic curves in the same clusters have mean infectious period distributions with a difference of one day. These observations suggest that the infectious period distributions have some influence on the clustering.

**Figure 9:**
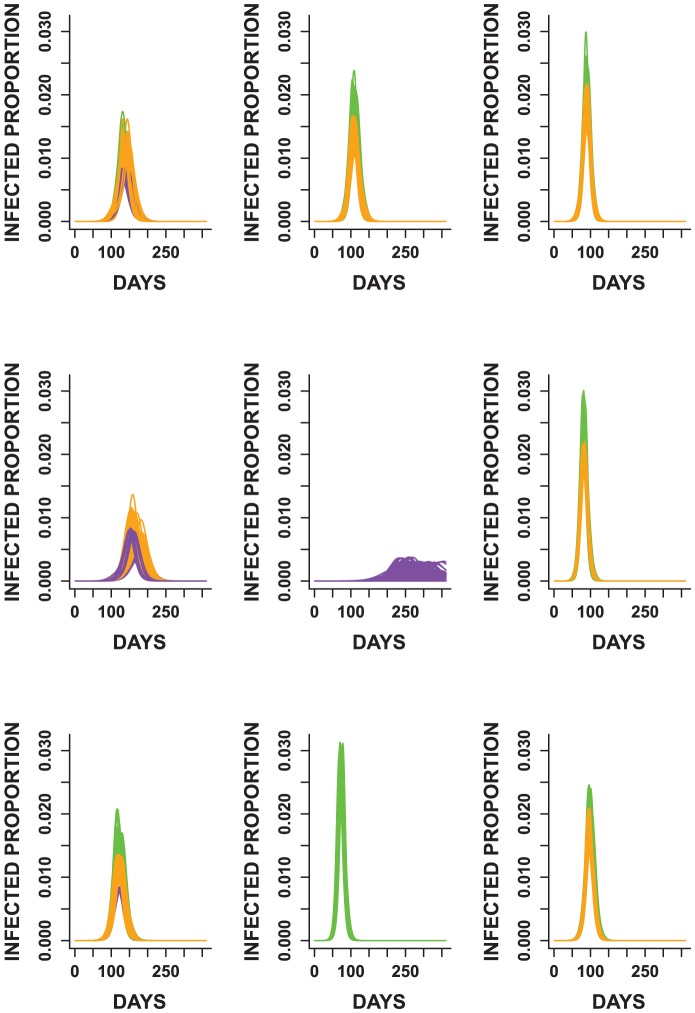
The epidemic curves from all 27 factorial experiments grouped using principal components cluster analysis. Note that curves in each cluster are grouped by time to peak and spread. Different colors are used to differentiate curves with different levels of the infectious period distribution. Green, orange and purple are used to represent epidemics with mean infectious period of five, four and three days respectively. In most cases, two levels of the infectious period are observed within each cluster.

The clustering in Figure 9 is color coded based on the levels of the infectious period distribution. Note that except for two clusters, all others have one or two levels of the infectious period distribution. In most clusters, we observe groupings of epidemic curves with mean infectious periods of three and four days or four and five days. In one cluster, all epidemics have a mean infectious period of five days. Furthermore, note that the curves in Figure 9 are grouped by peak time and spread, indicating that the clustering captured the structure of the epidemic curves.

We also visualized the clustering based on the levels of the transmissibility and incubation period distributions (Figures S5 and S6). Unlike the groupings observed in the levels of the infectious period distribution, most of the clusters contain all levels of the incubation period distribution and the transmissibility. This implies that there is no particular influence of these parameters on the shape of the epidemic curves. These results therefore allude to the idea that the infectious period distribution exerts the strongest impact on the total attack rate and shape of the epidemic curves.

### Finding 4. The model sensitivity is consistent across social networks with demographic and rural-urban differences

#### Support

The same trends are observed across all experiments for both Montgomery County and New York. Although Montgomery County and New York have both demographic and rural-urban differences, these differences are not apparent in the epidemic outcomes. In Table 5, we present the Pearson correlation coefficients indicating the similarities between the results observed over the two social networks. The columns are labeled based on the notations used in Table 2. Note the high correlations signifying that the trend observed in the results is almost identical in most situations. For example, the column for Exp.1 showed that an increase in the transmissibility on average results in a shorter peak time, an increase in the total and peak infected proportions. This is because increasing the transmission value increases the probability of infections in the population.

**Table 5:** Pearson correlation between the trends observed in the results for Montgomery County and New York.

		Experiments			
Measures	Exp.1	Exp.2	Exp.3	Exp.4	Exp.5
Total attack rate	1.000	0.965	0.983	0.479	0.993
Peak attack rate	0.996	0.991	0.987	0.211	0.995
Peak time	0.996	0.999	0.998	0.751	0.921

The labels of the experiments are based on the labeling used in Table 2. Experiments using the two social networks result in similar outcomes.

The lowest correlations are observed for Exp.4, where we study the effect of the mean and variance of the incubation period distribution on the three public health measures. The conclusions drawn from this set of analysis indicate that the incubation period distributions result in epidemics with similar peak proportion infected and total attack rates. There is no trend observed between the three public health measures and changes in the variances; therefore the lack of significant correlation. However, the overall observations are similar across the social networks. This therefore suggests that the results observed in this sensitivity study are not restricted to a particular network but can easily be applicable to others under certain assumptions.

### Finding 5. School-age children have the highest age-specific attack rates irrespective of mean infectious period and susceptibility of the other age groups

#### Support

**Figure 10:**
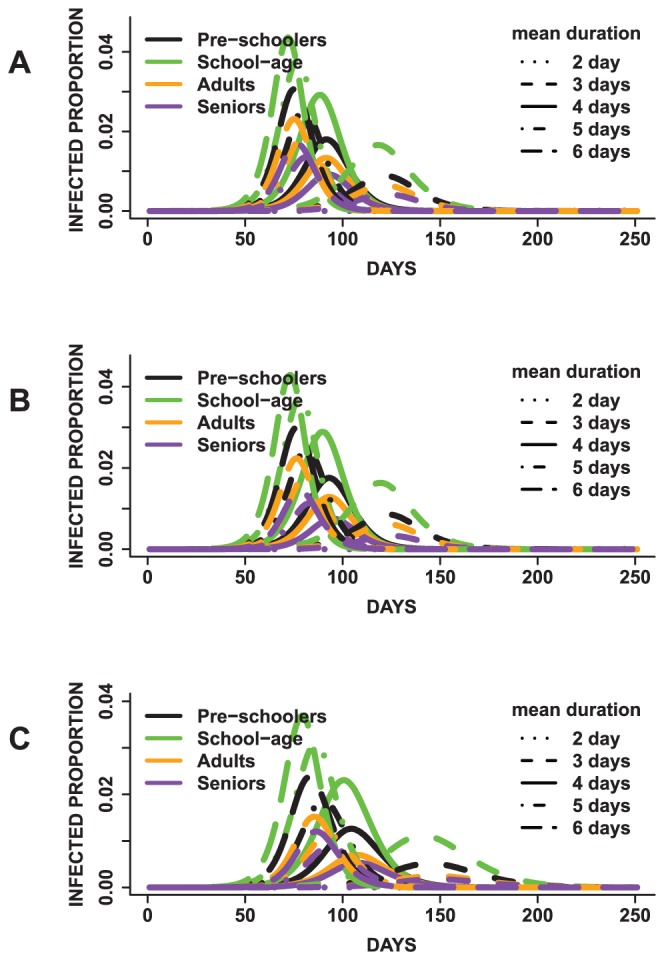
Age-specific mean epidemic curves presented by mean infectious duration. In (A), all age groups have the same disease susceptibility, (B) school-age children and elderly have a higher susceptibility and (C) school-age children have a higher susceptibility to the disease. Children have the highest mean peak infected proportion irrespective of the mean infectious period duration and susceptibility of the other age groups.

**Figure 11:**
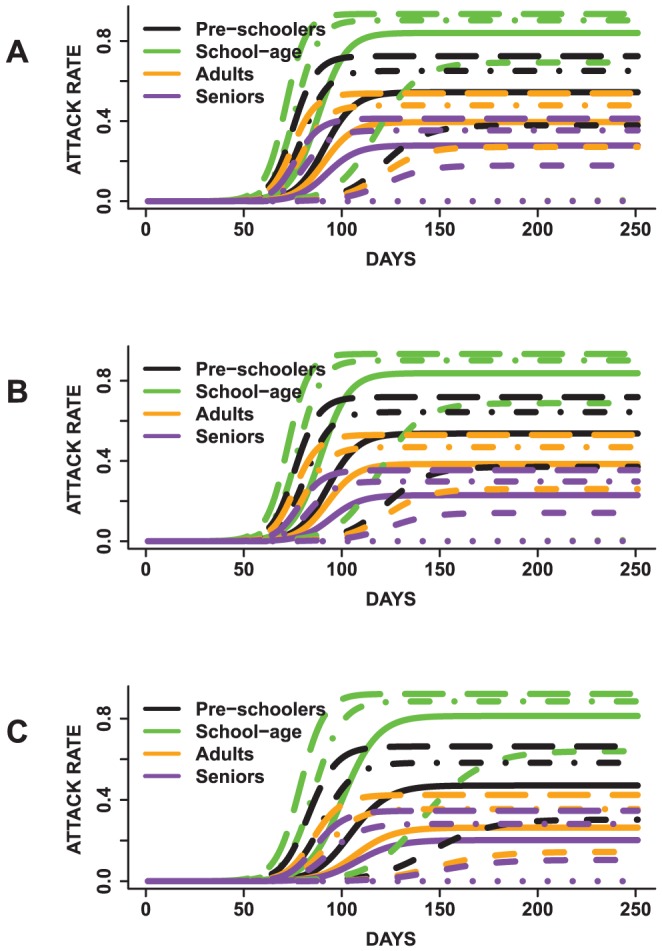
Age-specific mean total attack rates presented by mean infectious duration. In (A), all age groups have the same disease susceptibility, (B) school-age children and elderly have a higher susceptibility and (C) school-age children have a higher susceptibility to the disease. Children had the highest mean age-specific total attack rate across all epidemics irrespective of the mean infectious period duration and susceptibility of the other age groups.

In Figures 10 and 11, we display the mean epidemic curves for five sets of epidemics simulated under three scenarios. The five sets of epidemics are based on varying the mean infectious period between 2–6 days. Note that the outbreaks with mean infectious duration of two days are not displayed since the simulations failed to become epidemics. The three scenarios are based on: assigning the same disease susceptibility to all age groups (Figures 10(a) and 11(a)), assigning a higher susceptibility to school-age children and elderly (Figures 10(b) and 11(b)) and defining a higher susceptibility only for school-age children (Figure 10(c) and 11(c)). These will be called first, second and third scenarios for the remainder of this section. The discussions are focused on how these changes affect the school-age epidemics relative to the other groups.

School-age children have the highest age-specific total attack rates for all epidemics across all scenarios. Preschoolers have the second highest age-specific total attack rates. Adults have third highest age-specific total attack rates and the elderly have the lowest attack rates. For example, under the first scenario, epidemics for which the population have a mean infectious period of five days, school-agers, preschoolers, adults and elderly have a mean age-specific total attack rate of approximately *90%, 65%, 47%* and *35%* respectively. Similar attack rates are observed under the other two scenarios. Allowing school-age children to have a higher susceptibility compared to other age groups does not result in higher attack rates compared to the case in which all age groups have the same susceptibility. These findings suggest that neither increasing the mean infectious period nor changing the susceptibility affects the trend of disease spread observed across age groups. The high attack rates observed for school-age children could be attributed to the network degree and average contact time distributions. Children have a mean degree of 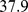 and average person contact time of 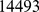 seconds. This is higher than the average degree of 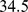 and contact time of 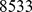 seconds observed for adults. Since children have a higher average contact time and tend to be more connected, they are also more likely to become infected.

The overall trend of disease spread is similar across all three scenarios. The trends observed in the attack rates and peak times are similar for all sets of epidemics and age groups 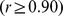. In addition, under the first scenario, the following pairs do not have statistical significantly different peak times for epidemics with mean infectious duration of three days: (preschoolers, seniors), (preschoolers, adults) and (adults, seniors) with 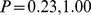 and *0.16* respectively. For epidemics with mean infectious duration of four days, we do not observe statistical significant differences in the following age group pairs: (preschoolers, seniors), (preschoolers, adults) and (adults, seniors) with *P = 0.183, 1.00* and *0.094* respectively. The same pairs do not have statistically different peak times (*P = 1.00*) for epidemics with mean infectious periods of five and six days. Note that the school-age population is not included in any of the pairs with similar peak times. On the contrary, all pairwise comparisons of the total attack rates resulted in statistically significant differences across all age groups and epidemics (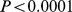). P-values of one are due to Bonferroni adjustment.

Per Figures 10 and 11, longer mean infectious periods result in shorter times to peak and higher attack rates. However, the total and peak infected proportions differ between scenarios for each epidemic. The attack rates for school-agers appear to be more practically similar between scenarios (1) and (2), although a pairwise comparison of the means indicate statistical significant differences (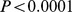). Epidemics with mean infectious period durations of five and six days seem to have more similar attack rates compared to epidemics with mean infectious duration of three and four days. Lastly, the peak time steadily shortened with an increase in the mean infectious period.

## Discussion

In this study, we performed sensitivity analysis on discrete probability distributions parameters for an individual-based model for influenza. The major findings in this study were: (i) minute changes in the disease parameters significantly increased the peak proportion infected, total attack rate and time to peak. (ii) Knowing the mean of the incubation period distribution appeared to be sufficient in predicting its effects on the dynamics of a simulated epidemic. (iii) The characteristics of the infectious period distribution affected the total attack rate and structure of the epidemic curves. (iv) The sensitivity of the individual-based model was independent of the demographical aspects of the social networks. (v) Differences in age-group susceptibility did not influence the overall trend of disease severity observed within the population. These observations can aid in improving real-time epidemic modeling using similar models.

Two important measures to predict during an epidemic are the time to peak and the total attack rate [25]. However, to make accurate predictions of these measures, we need good estimates of parameters such as the transmissibility, incubation and infectious period. Typically, these parameters are estimated using household transmission data [45]. These are usually observational studies and not experimental studies. Understanding that the models can be highly sensitive to small changes in the parameters can aid in mitigating some of the bias usually observed in observational studies. In addition, upon estimation of the parameters, intervention strategies can be evaluated to control the spread of the epidemic.

The uncertainty in real-time epidemic modeling and predictions in the early stages of an outbreak are partly due to the imperfection of incidence data [23]. However, incomplete knowledge of the effects of the parameters can also influence predicted outcomes. Since there is not a one-to-one mapping of epidemic characteristics to disease parameters, different parameter combinations can produce epidemics with similar characteristics. Therefore, both biological details on the virus and epidemiological data are needed to improve real-time epidemic modeling [23].

However, this study is not without limitations. The space explored for the parameters in the experiments was limited since in most cases, differences in the mean incubation and infectious period distributions lay between 1–3 days. More extreme differences might be observed if more extensive distributions were used. However, one can argue that using parameters which are similar provides a more strenuous analysis on the system, since parameters with significant influences would be more distinguishable from those with minute influences. In addition, assumptions about the disease model were simplified for this study. Additional layers can be included to describe different compartments of the population, such as infected asymptomatic and infected symptomatic individuals. Furthermore, future studies can also evaluate sensitivity in the presence of various intervention strategies and also with additional information such as school opening dates by region [46].

In addition, since network structure has been shown to influence disease dynamics [43], [47], [48], a study evaluating the joint effects of changes to the network structure and the disease parameters would also be beneficial.

The results in this study add to the growing literature of real-time modeling of epidemics [21], [24], [32], [33]. These methods are essential for pandemic planning and improving public policy decisions. In addition, this study provides a framework from which future studies can build on for more complex sensitivity analysis on individual-based models with discrete probability distribution parameters. Network models based on urban transportation systems, ad hoc communication and computing systems, and public health which use a probabilistic structure to describe interaction between nodes can all benefit from this study [49]. Furthermore, the level of sensitivity of this model to slight changes in these parameters reaffirms the idea that studies about epidemics using individual-based models are suggestions and not precise predictions, which could benefit public health pandemic planning.

## Supporting Information

Figure S1 **Results from mono-factorial experiments (Exp.4 in** **Table 2** **) focused on changes in the variance of the incubation period distribution.** (A) Plot of the variance of the incubation period distribution against the peak proportion infected and (B) plot of variance of the incubation period distribution against the peak time. No obvious pattern is observed in the relationship between the two measures and the incubation period variance.
(EPS)

Figure S2 **Results from mono-factorial experiments (Exp.5 in** **Table 2** **) focused on changes in the variance of the infectious period distributions.** (A) Plot of the variance of the infectious period distribution against the peak proportion infected and (B) figure of the variance of the infectious period distribution against the peak time. Note, the linear trend between the two measures and the variance of the infectious period.
(EPS)

Figure S3 **Mean epidemic curves from 50 replicates of each full factorial experiment (Exp.6 in** **Table 2** **).** Each outbreak is simulated for a duration of 350 days so as to accommodate outbreaks that are similar to previous influenza pandemics, seasonal epidemics and more extreme outbreaks. (A) shows the total proportion of the population infected over time. (B) is the daily proportion of infected individuals. Although the parameters used in producing the epidemics are different, the resulting dynamics are similar for some of the epidemics.
(EPS)

Figure S4 **The epidemic curves from all 27 factorial experiments are grouped using principal components cluster analysis.** Note that curves in each cluster are grouped by peak time and spread. Different colors are used to differentiate curves resulting from replicates of each of the 27 epidemics. Epidemic curves from all experiments appear to be randomly distributed across clusters, irrespective of parameter combinations.
(EPS)

Figure S5 **The epidemic curves from all 27 factorial experiments are grouped using principal components cluster analysis.** Different colors are used to differentiate curves from different transmissibility levels. Unlike the groupings observed in the levels of the infectious period distribution (Figure 9), no apparent groupings of factor levels were observed for the transmissibility.
(EPS)

Figure S6 **The epidemic curves from all 27 factorial experiments are grouped using principal components cluster analysis.** Different colors are used to differentiate curves from different incubation period distributions. Unlike the groupings observed in the levels of the infectious period distribution (Figure 9), no obvious groupings of factor levels were observed for the incubation period distribution.
(EPS)

Figure S7
**The effective reproduction number R(t) for mono-factorial experiments for each of the factors: (A) transmissibility, (B) incubation period distribution and (C) infectious period distribution.** Each curve represents the mean effective reproduction number based on 25 replicates of each experiment. (A) shows the effective reproduction number given changes in the transmissibility from 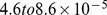 per sec/contact time, (B) illustrates the effective reproduction as the mean of the incubation period distribution changes between 2 to 6 days and (C) is the effective reproduction as the mean of the infectious period distributions varies between 1 and 5 days.(EPS)

Figure S8 **Degree distribution for children (A) and adults (B) in the NY social network.**The long tails are consistent with what one would expect in the real world. The higher mean degree observed in children might be the reason for the higher age-specific attack rates observed in children.
(EPS)

Model S1 **Description of the individual-based model used in this study.**
(TEX)

Table S1 **Analysis of contributions of the parameters to the variance observed in the total attack rate, peak proportion infected, and peak time.** The first row shows the total variance in each of the three outcome measures, while the rows beneath display the fraction of the total variance explained by each factor. t is used to represent transmissibility, inc abbreviates incubation period distribution and infc represents the infectious period distribution. The infectious period distribution explains the highest proportion of the variation observed in all three measures.
(TEX)

## References

[1] Saltelli A, Annoni P, Azzini I, Campolongo F, Ratto M, et al (2010) Variance based sensitivity analysis of model output. Design and estimator for the total sensitivity index. Computer Physics Communications 181: 259–270.

[2] Helton JC (2008) Uncertainty and Sensitivity Analysis for Models of Complex Systems. In: Graziani F, editor, Computational Methods in Transport: Verification and Validation, Springer Berlin Heidelberg, volume 62 of Lecture Notes in Computational Science and Engineering, chap-ter 9. pp. 207–228.

[3] Cacuci DG, Ionescu-Bujor M, Navon IM (2005) Sensitivity And Uncertainty Analysis: Applications to Large-Scale Systems. New York: Chapman & Hall/CRC, volume ii edition.

[4] Ginot V, Gaba S, Beaudouin R, Aries F, Monod H (2006) Combined use of local and anova-based global sensitivity analyses for the investigation of a stochastic dynamic model: application to the case study of an individual-based model of a fish population. Ecological Modelling 193: 479491.

[5] Frey HC, Patil SR (2002) Identification and Review of Sensitivity Analysis Methods. Risk Analysis 22: 553–578. 12088234

[6] Hamby DM (1995) A comparison of sensitivity analysis techniques. Health Physics 68: 195–204. 7814253 10.1097/00004032-199502000-00005

[7] Saltelli A, Tarantola S, Campolongo F, Ratto M (2004) Sensitivity Analysis in Practice: A Guide to Assessing Scientific Models. New York, NY, USA: Halsted Press.

[8] Epstein J (2009) Modelling to contain pandemics. Nature 687. 10.1038/460687a PMC3785367 19661897

[9] Longini I, Nizam A, Xu S, Ungchusak K, Hanshaworakul W, et al (2005) Containing pandemic inuenza at the source. Science 309: 1083–1087. 16079251 10.1126/science.1115717

[10] Chao DL, Halloran ME, Obenchain VJ, Longini IM (2010) FluTE, a Publicly Available Stochastic Inuenza Epidemic Simulation Model. PLoS Computational Biology 6: e1000656+. 20126529 10.1371/journal.pcbi.1000656 PMC2813259

[11] Barrett C, Bisset K, Leidig J, Marathe A, Marathe M (2011) Economic and social impact of inuenza mitigation strategies by demographic class. Epidemics 3: 19–31. 21339828 10.1016/j.epidem.2010.11.002 PMC3039122

[12] Bisset K, Chen J, Feng X, Kumar VSA, Marathe M (2009) Epifast: a fast algorithm for large scale realistic epidemic simulations on distributed memory systems. In: Proceedings of the 23rd international conference on Supercomputing. ICS '09, pp. 430–439.

[13] Heath B, Hill R, Ciarallo F (2009) A survey of agent-based modeling practices (january 1998 to july 2008). Journal of Artificial Societies and Social Simulation 12: 9.

[14] Burke D, Epstein J, Cummings D, Parker J, Cline K, et al (2006) Individual-based Computational Modeling of Smallpox Epidemic Control Strategies. Academic Emergency Medicine 13: 1142–1149. 17085740 10.1197/j.aem.2006.07.017

[15] Halloran ME, Ferguson N, Eubank S, Longini I, Cummings D, et al (2008) Modeling targeted layered containment of an inuenza pandemic in the United States. Proceedings of the National Academy of Sciences 10.1073/pnas.0706849105 PMC2290797 18332436

[16] Germann TC, Kadau K, Longini IM, Macken CA (2006) Mitigation strategies for pandemic in-uenza in the United States. Proceedings of the National Academy of Sciences 103: 5935–5940. 10.1073/pnas.0601266103 PMC1458676 16585506

[17] Beckman R, Baggerly K, Mckay M (1996) Creating synthetic baseline populations. Transportation Research Part A: Policy and Practice 30: 415–429.

[18] Eubank S, Guclu H, Kumar VSA, Marathe M, Srinivasan A, et al (2004) Modelling disease out-breaks in realistic urban social networks. Nature 10.1038/nature02541 15141212

[19] Eubank S, Barrett C, 650 Beckman R, Bisset K, Durbeck L, et al (2010) Detail in network models of epidemiology: are we there yet? Journal of Biological Dynamics 4: 446–455. 20953340 10.1080/17513751003778687 PMC2953274

[20] Clinical Signs and Symptoms of Inuenza. Available: http://www.cdc.gov/flu/professionals/acip/clinical.htm. Accessed 2012 Aug 27.

[21] Nsoesie EO, Beckman R, Marathe M, Lewis B (2011) Prediction of an epidemic curve: A supervised classification approach. Statistical Communications in Infectious Diseases 3. 10.2202/1948-4690.1038 PMC3445421 22997545

[22] Goldstein E, Apolloni A, Lewis B, Miller J, Macauley M, et al (2010) Distribution of vac-cine/antivirals and the “least spread line” in a stratified population. Journal of the Royal Society Interface 7: 755–764. 10.1098/rsif.2009.0393 PMC2874227 19828505

[23] Lipsitch M, Finelli L, Heffernan RT, Leung GM, Redd SC (2011) Improving the evidence base for decision making during a pandemic: the example of 2009 inuenza A/H1N1. Biosecurity and bioterrorism biodefense strategy practice and science 9: 89–115. 10.1089/bsp.2011.0007 PMC3102310 21612363

[24] Ong JBS, Chen MIC, Cook AR, Lee HC, Lee VJ, et al (2010) Real-time epidemic monitoring and forecasting of H1N1-2009 using inuenza-like illness from general practice and family doctor clinics in singapore. PLoS ONE 5: e10036. 20418945 10.1371/journal.pone.0010036 PMC2854682

[25] Chao DL, Matrajt L, Basta NE, Sugimoto JD, Dean B, et al (2011) Planning for the control of pandemic inuenza A (H1N1) in los angeles county and the united states. American Journal of Epidemiology 173: 1121–1130. 21427173 10.1093/aje/kwq497 PMC3121321

[26] Opatowski L, Fraser C, Griffin J, De Silva E, Van Kerkhove MD, et al (2011) Transmission char-acteristics of the 2009 H1N1 inuenza pandemic: Comparison of 8 southern hemisphere countries. PLoS Pathogens 7. 10.1371/journal.ppat.1002225 PMC3164643 21909272

[27] Merler S, Ajelli M (2010) The role of population heterogeneity and human mobility in the spread of pandemic inuenza. Proceedings of the Royal Society B: Biological Sciences 277: 557–565. 19864279 10.1098/rspb.2009.1605 PMC2842687

[28] Monto AS, Davenport FM, Napier JA, Francis T Jr (1969) Effect of vaccination of a school-age population upon the course of an A2/Hong Kong inuenza epidemic. Bull World Health Organ 41: 53742. PMC2427727 5309469

[29] Reichert TA, Sugaya N, Fedson DS, Glezen WP, Simonsen L, et al (2001) The Japanese Experience with Vaccinating Schoolchildren against Inuenza. New England Journal of Medicine 344: 889–896. 11259722 10.1056/NEJM200103223441204

[30] Salathé M, Kazandjieva M, Lee JW, Levis P, Feldman MW, et al (2010) A high-resolution hu-man contact network for infectious disease transmission. Proceedings of the National Academy of Sciences 10.1073/pnas.1009094108 PMC3009790 21149721

[31] Basta NE, Chao DL, Halloran ME, Matrajt L, Longini IM (2009) Strategies for pandemic and seasonal inuenza vaccination of schoolchildren in the united states. American Journal of Epi-demiology 170: 679–686. 10.1093/aje/kwp237 PMC2737588 19679750

[32] Nishiura H (2011) Real-time forecasting of an epidemic using a discrete time stochastic model: a case study of pandemic inuenza (H1N1-2009). Bio Medical Engineering Online 10. 10.1186/1475-925X-10-15 PMC3045989 21324153

[33] Ohkusa Y, Sugawara T, Taniguchi K, Okabe N (2011) Real-time estimation and prediction for pandemic A/H1N1(2009) in Japan. Journal of infection and chemotherapy 10.1007/s10156-010-0200-3 21387184

[34] Barrett CL, Beckman R, Berkbigler K, Bisset K, Bush K, et al.. (2001) TRANSIMS: Transportation analysis simulation system. Technical Report, LA-UR-00-1725, Los Alamos National Laboratory Unclassified Report 3.

[35] Speckman P, Vaughn K, Pas E (1997a) Generating household activity-travel patterns (HATPs) for synthetic populations. Transportation Research Board 1997 Annual Meeting.

[36] Speckman P, Vaughn K, Pas E (1997b) A continuous spatial interaction model: Application to home-work travel in Portland, Oregon. Transportation Research Board 1997 Annual Meeting.

[37] Bowman J (2001) Activity-based disaggregate travel demand model system with activity schedules. Transportation Research Part A: Policy and Practice 35: 1–28.

[38] Bowman J, Bradley M, Shiftan Y, Lawton TK, Ben-Akiva M (1998) Demonstration of an activity based model system for Portland. In: Proceedings of the 8th World Conference on Transport Research.

[39] Yang Y, Halloran ME, Daniels MJ, Longini IM, Burke DS, et al (2010) Modeling competing infectious pathogens from a bayesian perspective: Application to inuenza studies with incomplete laboratory results. Journal of the American Statistical Association 1310–1322. 21472041 10.1198/jasa.2010.ap09581 PMC3070363

[40] McKay MD, Beckman RJ, Conover WJ (1979) A comparison of three methods for selecting values of input variables in the analysis of output from a computer code. Technometrics 21: 239245.

[41] Box GEP, Hunter WG, Hunter JS, Hunter WG (1978) Statistics for Experimenters: An Introduc-tion to Design, Data Analysis, and Model Building. John Wiley & Sons.

[42] Beckman R, Chaturvedi G, Lewis B (2008) Clustering principal components to _nd groupings in a collection of curves. Technical Report 07-043, NDSSL, VBI, Virginia Tech, Blacksburg, Virginia.

[43] Rahmandad H, Sterman J (2008) Heterogeneity and network structure in the dynamics of diffusion: Comparing agent-based and differential equation models. Management Science 54: 998–1014.

[44] Cowling BJ, Chan KH, Fang VJ, Lau LLH, So HC, et al (2010) Comparative Epidemiology of Pandemic and Seasonal Inuenza A in Households. New England Journal of Medicine 362: 2175–2184. 20558368 10.1056/NEJMoa0911530 PMC4070281

[45] Cauchemez S, Donnelly CA, Reed C, Ghani AC, Fraser C, et al (2009) Household transmission of 2009 pandemic inuenza A (H1N1) virus in the United States. New England Journal of Medicine 361: 2619–2627. 20042753 10.1056/NEJMoa0905498 PMC3840270

[46] Chao DL, Halloran ME, Longini IM (2010) School opening dates predict pandemic inuenza a(h1n1) outbreaks in the united states. Journal of Infectious Diseases 202: 877–880. 20704486 10.1086/655810 PMC2939723

[47] Keeling MJ, Eames KT (2005) Networks and epidemic models. Journal of the Royal Society, Interface/the Royal Society 2: 295–307. 10.1098/rsif.2005.0051 PMC1578276 16849187

[48] Pautasso M, Jeger MJ (2008) Epidemic threshold and network structure: The interplay of proba-bility of transmission and of persistence in small-size directed networks. Ecological Complexity 5: 1–8.

[49] Barrett C, Eubank S, Kumar V, Marathe M (2004) Understanding large scale social and infras-tructure networks: A simulation based approach. SIAM news: The Mathematics of Networks

